# Assessment of knowledge and implementation practices of the ventilator acquired pneumonia (VAP) bundle in the intensive care unit of a private hospital

**DOI:** 10.1186/s13756-021-01027-1

**Published:** 2021-11-12

**Authors:** Cybele Lara Abad, Cordella P. Formalejo, Dan Meynard L. Mantaring

**Affiliations:** 1 Division of Infectious Diseases, Department of Medicine, University of the Philippines Manila, Philippine General Hospital, Manila, Philippines; 2 Section of Infectious Diseases, Department of Medicine, The Medical City, Ortigas Avenue, Pasig, Philippines; 3 Department of Medicine, The Medical City, Ortigas Avenue, Pasig, Philippines

**Keywords:** Adherence, Bundles of Care, ICU, Infection control, Knowledge, VAP

## Abstract

Studies have shown that a ventilator associated pneumonia (VAP) bundle significantly decreases VAP rates. In this study, we evaluated existing knowledge, practices, and adherence of nurses and infection control preventionists (ICP) to the VAP bundles of care in the intensive care unit (ICU) by using qualitative and quantitative tools. Of 60 participants (56 nurses and 4 ICPs), mean knowledge score regarding specific evidence-based VAP guidelines was 5 (range 3–8) out of 10 points. Self-reported adherence to the VAP bundle ranged from 38.5 to 100%, with perfect compliance to head of bed elevation, and poorest compliance with readiness to extubate. Overall VAP median bundle compliance was 84.6%. Knowledge regarding specific components of VAP prevention is lacking. Formal training and interactive educational sessions should be performed regularly to assess the competency of key personnel regarding the VAP bundle, especially in the context of rapid nurse turnover. Incentives for retention of nurses should also be considered, so that knowledge of hospital specific initiatives such as the VAP bundles of care can be cultivated over time.

**Supplementary Information:**

The online version contains supplementary material available at 10.1186/s13756-021-01027-1.

## Background

The Institute of Healthcare Improvement (IHI) has advocated the use of “Ventilator Associated Pneumonia (VAP) Bundles of Care” to decrease morbidity and mortality in patients with VAP. The bundle components include head of bed elevation between 30° and 45°, closed suctioning and subglottic drainage, daily assessment of readiness to extubate, deep venous thrombosis (DVT) prophylaxis, and oral care. IHI estimates that over 122,000 lives have been saved, with decreased days of mechanical ventilation and hospital stay, through this initiative [1]. However, a greater than 95% compliance rate with the VAP bundle is often needed to achieve zero incidence of VAP [2] and periodic assessment of the medical and nursing staff is often recommended to improve long-term compliance [3]. A local study showed that implementation of the VAP bundle in The Medical City (TMC) decreased VAP rates [4]. Despite its use, however, VAP remains the most common hospital acquired infection (HAI) in the TMC-intensive care unit (ICU), with rates as high as 7.92 per 1000 ventilator days in 2017. This study aimed to assess the knowledge, adherence, and practices of key healthcare personnel regarding VAP prevention strategies and the VAP bundle.

## Methods

ICU nurses and infection control preventionists (ICP) were informed about the study through a letter addressed to the Hospital Infection Control and Epidemiological Center (HICEC), Acute Critical Care Institute (ACCI) and Institute of Neurological Sciences (INS). The HICEC is comprised of 5 ICPs, and both the ACCI and INS delegate a total of 89 nursing staff to the ICU complex. After study approval by the Institutional Review Board (TMC IRB 2019), all ICU nursing and ICP staff were formally invited using hospital communications (e.g. hospital intranet, announcement during ICU staff meetings), and informed consent acquired. We undertook a descriptive study using both quantitative and qualitative methods, as described below.

### Survey and small group discussion

Two sets of questionnaires (Additional file 1: "Knowledge of EBG on VAP Prevention" Appendix Part I) were given to both the ICU nurses and ICPs. Each participant was expected to respond, and given a randomly assigned number as indicated in the questionnaires.

The first questionnaire evaluated knowledge of evidence-based guidelines on VAP prevention. A modified version [5] of a pre-validated ventilator bundle questionnaire (VBQ) based on a study done in 2007 by Labeau et al. was used [6]. It was comprised of ten objective closed-ended questions, to measure the participants’ level of knowledge. One point was given for each correct answer, with scores ranging from 0 to 10.

A second questionnaire was used to determine the self-reported adherence to the TMC VAP bundle, and the barriers encountered in its implementation in the ICU. A modified version of Jansson et al*.*’s VBQ [5], based on an earlier study [7] was used, with only 13 of the original 25 strategies relevant to the TMC VAP bundle included. One point was given for adherence to each item. If any item was not adhered to, the participants were asked to indicate 1 of 8 predefined barriers to the component’s implementation (Additional file 1: "Self-reported Adherence to the VAP Bundle" Appendix Part II).

In a separate session, thirty minutes was allotted for small group discussion (SGD). All participants were invited, and the SGD was carried out in three sub-groups, in a conference room within the hospital, during pre-duty nursing shifts. During this period, the participants’ knowledge, adherence to, and skills regarding the VAP bundle were evaluated using pre-determined questions. The SGD was recorded using a mobile device app and transcribed by study authors (DMM, CPF)*.*

### Direct observation

The primary investigators (DMM, CFP) directly observed ICU nurses for three non-consecutive days to determine compliance to the VAP bundle. The observation period was usually within the 6 am to 6 pm shift, with compliance to each bundle element observed for about 3–5 min, totaling 16 different observation events. Both DMM and CFP were medical residents (e.g. part of the ICU healthcare team) at this time, and the nurses were unaware that they were being monitored as part of the study to eliminate bias in determining adherence to the VAP bundles. Apart from direct observation, charts of mechanically ventilated patients in the ICU complex were reviewed to evaluate compliance to specific components (e.g. DVT prophylaxis, oral care and readiness to extubate) by using a checklist (Additional file 1: "VAP Bundle Compliance Checklist", Appendix 2). Compliance to each component of the bundle incurred one [1] point. Mean compliance was then determined per item of the bundle. The primary investigators then compared results with the TMC 2017 HICEC’s VAP bundle compliance data.

We used descriptive statistics and determined frequency distributions of demographic and clinical characteristics for quantitative variables. We used median as our main measure of central tendency in a small patient population with small and large values. Mean was used for knowledge scores given the small values (e.g. 1–10). To analyze, categorize, and quantify the answers to the open-ended questions used to identify barriers to VAP bundle implementation, all answers to the questions were collected in subcategories based on the respondents’ descriptions using open coding.

## Results

### Characteristics of the study population

Of the 89 nurses in the ICU Complex and five (5) ICPs, 60 (56 nurses, 4 ICPs) (64%) consented to take part in the study. Among the 56 nurses, 43 (17 males, 26 females) were assigned in the main ICU (ICU/Telemetry). The remaining 13 nurses, of whom nine (9) were female, were assigned to the Acute Stroke Unit (ACSU). A summary of the respondents’ basic demographics, educational attainment, and clinical experience are summarized in Table 1. More than half (33/56, 58.98%) stated they had no formal training regarding the VAP bundle of care. Those who had training (21/33, 63.63%) were those had been assigned to the ICU for at least 4 years.

**Table 1:** Characteristics of the study population

Variables		ICP (n = 4), %	Nurses (n = 56), %
Age, median (range)		30 (26–35)	26 (21–48)
Gender, n (%)	Male	3 (75)	21 (37.5)
	Female	1 (25)	35 (62.5)
Work experience, # years (%)	1–5	3 (75)	42 (75)
	6–10	1 (25)	10 (18)
	> 10	0	4 (0.07)
Education (%)	BS	4 (100)	55 (98.21)
	MS	0	1 (1.79)
VAP bundle training (%)	Yes	1 (25)	23 (41.07)
	No	3 (75)	33 (58.93)

*ICP* Infection control practitioner, *BS* Bachelor of Science, *MS* Masters in Science

### Knowledge of the VAP bundle

Majority (44/60, 73.33%) of ICU nurses and ICPs were able to answer half of the questions correctly. Questions regarding frequency of ventilator circuit changes, type of airway humidifier, frequency of humidifier changes, open vs. closed suctioning system, and frequency of changes in suction systems were answered correctly only 13.3, 36.7, 21.7, 15.0 and 10.0% of the time. Questions regarding endotracheal intubation, subglottic suctioning, bed positioning, and use of chlorhexidine were answered correctly most of the time (Table 2).

**Table 2:** Knowledge regarding VAP bundles of care

Question	Correct response N = 60 (%)
1. Oral versus nasal route for endotracheal intubation	50 (83.3)
2. Frequency of ventilator circuit changes (*no response, n* = 1)	8 (13.3)
3. Type of airway humidifier (*no response, n* = 2)	22 (36.7)
4. Frequency of humidifier changes (*no response, n* = 3)	13 (21.67)
5. Open versus closed suction systems	9 (15)
6. Frequency of change in suction systems (*no response, n* = 1)	6 (10)
7. Endotracheal tubes with extra lumen for drainage of subglottic secretions	54 (90)
8. Kinetic vs. standard beds	44 (73.3)
9. Patient positioning (*no response, n* = 1)	56 (93.3)
10. Use of 0.12% chlorhexidine gluconate antiseptic oral rinse	58 (96.7)

### Adherence to the VAP bundle

Self-reported adherence ranged from 38.46 to 100%. Adherence to the VAP bundle as a whole was a median of 84.6%. All participants reported adherence to 30°-45° head of bed elevation. The component with the least adherence was performance of the spontaneous breathing test (32/60, 53.3%) (Table 3).

**Table 3:** Summary of adherence and barriers to the VAP bundles of care

Item	# Adhered (%)	Barriers							
		Disagreement with reported trial results	Inadequate resources	Fear of potential adverse effects	Costs	Patient discomfort	Lack of education	Lack of guidelines	Other comments
1. I always comply with the TMC VAP bundle	58 (96.7)		1			1			
2. I interrupt continuous sedative infusions as recommended	54 (90)		1			2	2	2	*Not scope of work*
3. I adhere to existing oral care protocol	59 (98.3)		1						
4. I always use chlorhexidine oral rinse as recommended	57 (95)					1			*Individualized care*
5. I always perform subglottic suctioning as recommended	54 (90)			1		1			*Unavailability and some do not have subglottic suction; not able to suction all*
6. I always use closed suction system for all my patients	55 (91.7)	1	1	1	1				*Unavailability*
7. I assess the depth of sedation as often as recommended	57 (95)								
8. I interrupt continuous sedative infusions as recommended	54 (90)					2	2		
9. I assess the depth of sedation using a validated tool	59 (98.3)								*Beyond nursing care*
10. I perform spontaneous breathing test as recommended	32 (53.3)			5			1	5	*Task of RT*
11. I always keep head of bed elevated at 30–45 degrees	60 (100)								
12. I always make sure that mechanical DVT prophylaxis are used as recommended	48 (80)		6	1			1	1	*Depends on patient condition*
13. I always give pharmacological DVT prophylaxis, as recommended	45 (75)								*Depends on patient condition*

Through direct observation, five out of the seven VAP bundle components were strictly followed—head of bed elevation, subglottic secretion drainage, use of closed suctioning system, oral care, and color-coding. These were consistent with HICEC data from 2017. Daily assessment of readiness to extubate was the component with the least compliance (68.7%). (Table 4).

**Table 4:** Direct observation at the intensive care unit

VAP bundle component	Direct observation (N = 16)	TMC 2017 (%)
Head of bed elevation	16	100
Subglottic secretion drainage	16	100
Daily assessment of readiness to extubate	11	34.6
Use of DVT prophylaxis	12	71.5
Use of closed suctioning system	16	100
Oral care	16	100
Color coding	16	100

### The VAP bundle in practice: small group discussion

#### Infection control practitioners

Almost all ICPs were able to identify the components of the bundles of care utilized in the ICU. According to the ICPs, VAP bundle training was done twice a year, and used as a prerequisite to become an ICP. However, ICPs were not required to attend a refresher course. Active participation (e.g. demonstration-return-demonstration) or exams were not included, and there was no formal evaluation of the ICP’s knowledge or skills. Among the seven components of TMC’s VAP bundle, they felt that they needed more education on the readiness to extubate. All ICPs agreed that all elements of the bundle needed to be performed, in order to be fully adherent with the VAP bundle.

#### ICU nurses

The nurses reported that VAP bundle training was given as part of a lecture series on the various bundles of care employed in the hospital; however, only a brief rationale of each VAP bundle component was given during the lecture, and a practical demonstration of each component was not done during training. A few were unaware that VAP bundle training was part of pre-employment training provided by the nursing service office. Those who experienced a Joint Commission International (JCI) accreditation mentioned that refresher courses on the VAP bundle were usually given only in preparation for re-accreditation. When asked regarding the sufficiency of knowledge on the VAP bundle, they felt they needed more thorough lectures and demonstrations. Only a few were aware that their compliance was monitored by HICEC. They were also unaware of feedback regarding their compliance rates.

## Discussion

Our study describes the knowledge, adherence and practices of nurses and ICPs regarding the VAP bundle. We highlight several key findings: first, the existence of a knowledge gap among both nurses and ICP regarding VAP prevention strategies; second, poor adherence to specific components of the VAP bundle, and third, lack of education and formal training were identified as the main barriers to VAP bundle adherence.

In general, all participants had difficulty answering the knowledge questionnaire, with a mean of 5.25 (range 3–8) points. Most correct responses were on questions directly related to nursing care (e.g., bed positioning, suctioning of secretions, and oral antisepsis). Questions regarding the use of airway humidifiers and frequency of ventilator circuit changes were incorrectly answered most of the time. One possible explanation for this, also observed in a prior study [8], is that other healthcare personnel such as respiratory therapists are assigned to maintain, assess, and care for humidifiers and ventilator tubings. As a result, the ICU nurses are less familiar with their maintenance. This suggests that components of the VAP bundle related to airway and ventilation should be frequently taught to nursing staff in the ICU to address this specific knowledge gap.

Our nurses with longer experience in the ICU (i.e. > 4 years) were more likely to have undergone VAP bundle training. However, their knowledge (score of 6 points) appeared similar to less experienced nurses (data not shown), which differs from published data [6]. In one study [8] for example, the average knowledge level was higher among more experienced ICU nurses (> 1 year experience) and those holding a special degree in emergency and intensive care. Retention of ICU nurses so they gain experience is particularly problematic in private hospitals in the Philippines where turnover is high because of lower wages, fewer opportunities for career development, better financial incentives abroad, and the prospect of migration [9]. Consequently, providing incentives for both recruitment and long-term retention of these nurses have to be addressed, so that knowledge of hospital specific initiatives such as the VAP bundles of care is cultivated over time.

Majority of respondents reported high rates of self-adherence with the VAP bundle—head of bed elevation was easiest to comply with, while spontaneous breathing trials and DVT prophylaxis were the most difficult. The reluctance to perform breathing trials was related to fear of potential adverse events such as precipitating patient discomfort or shortness of breath [10], as well as lack of training and specific guidelines. Similarly, compliance with DVT prophylaxis was not uniformly followed. We speculate that this modest compliance with the bundle components is rooted in lack of formal training, and absence of specific guidance regarding implementation, as the SGD highlighted the same issues—informal handover of knowledge, and irregular or inconsistent training.

The lack of education and training were consistently identified as the principal reasons precluding proper implementation of the VAP bundle. Several studies have shown the impact of educational interventions. In a 2-year study among healthcare workers in a 20 bed medical intensive care unit (MICU) [11], for example, educational programs and reminders were implemented to increase compliance to VAP prevention strategies. A 3-h mandatory slide presentation on the epidemiology and pathophysiology of VAP and preventive measures was given to the MICU healthcare workers, followed by an interactive discussion. This presentation was repeated for new employees. Reminders were also displayed on the MICU computer screensavers. The median composite score throughout the study (baseline, 1 month, 6 months, 12 months and 24 months) significantly increased after continuous implementation of the program for all healthcare workers in the ICU (*p* < 0.0001) from a baseline of 2, to as high as 5 points, over time. The rate of VAP decreased by 51%, after the intervention (*p* < 0.0001) [11]. The success of this intervention highlights the importance of regular and consistent education and feedback, which is currently lacking in our institution.

Our study has several limitations. Our sample size was small and not all ICU nurses participated in the study. Our assessment of knowledge was based solely on one validated questionnaire, and our period of direct observation was limited. We also did not directly co-relate VAP bundle adherence with VAP rates. Nevertheless, this is the first study to determine baseline knowledge, adherence, and implementation practices of key personnel directly involved with implementation of the VAP bundle.

## Conclusion

We find that both ICP’s and nurses are aware of the VAP bundle. Compliance to the bundle as a whole is modest, and knowledge of key components of VAP prevention guidelines is still lacking. We advocate that formal training and interactive educational sessions be done regularly to assess the competency of key personnel regarding the VAP bundle, especially in the context of rapid nurse turnover. Incentives for retention of nurses should also be strongly considered, so that knowledge of hospital specific initiatives such as the VAP bundles of care can be cultivated over time.

## Supplementary Information

**Additional file 1.** Ventilator bundle questionnaire to assess knowledge of evidence-based guidelines on VAP prevention (Part 1) and self-reported adherence and barriers to the VAP bundle (Part 2). VAP bundle checklist showing compliance (or non-compliance) with each bundle element.

## Data Availability

The datasets used and/or analyzed during the current study are available from the manuscript and supplementary data.

## Abbreviations

ACCI: Acute and Critical Care Institute
ACSU: Acute Stroke Unit
DVT: Deep Venous Thrombosis
HAI: Hospital Acquired Infection
HICEC: Hospital Infection Control and Epidemiological Center
ICP: Infection Control Preventionist
ICU: Intensive Care Unit
IHI: Institute of Healthcare Improvement
INS: Institute of Neurological Science
IRB: Institutional Review Board
JCI: Joint Commission International
MICU: Medical Intensive Care Unit
PSMID: Philippine Society of Microbiology and Infectious Diseases
RT: Respiratory Therapist
TMC: The Medical City
VAP: Ventilator Acquired Pneumonia
