# Supplementary material for: Assessment of knowledge and implementation practices of the ventilator acquired pneumonia (VAP) bundle in the intensive care unit of a private hospital
Source: Antimicrob Resist Infect Control. 2021 Nov 12;10:161. doi: 10.1186/s13756-021-01027-1 (PMC8587499; doi:10.1186/s13756-021-01027-1)
Supplement: Supplementary file 1 — Additional file 1. Ventilator bundle questionnaire to assess knowledge of evidence-based guidelines on VAP prevention (Part 1) and self-reported adherence and barriers to the VAP bundle (Part 2). VAP bundle checklist showing compliance (or non-compliance) with each bundle element. [file 13756_2021_1027_MOESM1_ESM.docx]

**Supplemental Appendix 1.**

**VENTILATOR BUNDLE QUESTIONNAIRE FOR HICEC AND ICU NURSES**

**PART I: Knowledge of Evidence-Based Guidelines (EBGs) on VAP Prevention**

Number:________ Job description: RN_____ Infection Preventionist_____

Age/Sex:_______ Years in service:______________ Education:___________________________________________

Have you every had training or seminars regarding EBGs on VAP prevention? Y__ N__

*Instructions: Encircle the letter corresponding to your answer.*

1. Oral vs. nasal route for endotracheal intubation
   1. Oral intubation is recommended
   2. Nasal intubation is recommended
   3. Both routes of intubation can be recommended
   4. I do not know
2. Frequency of ventilator circuits changes
   1. It is recommended to change circuits every 48 hours (or when clinically indicated)
   2. It is recommended to change circuits every week (or when clinically indicated)
   3. It is recommended to change circuits for every new patient (or when clinically indicated)
   4. I do not know
3. Type of airway humidifier
   1. Heated humidifiers are recommended
   2. Heat and moisture exchangers are recommended
   3. Both types of humidifiers can be recommended
   4. I do not know
4. Frequency of humidifier changes
   1. It is recommended to change humidifiers every 48 hours (or when clinically indicated)
   2. It is recommended to change humidifiers every 72 hours (or when clinically indicated)
   3. It is recommended to change humidifiers every week (or when clinically indicated)
   4. I do not know
5. Open vs. closed suction systems
   1. Open suction systems are recommended
   2. Closed suction systems are recommended
   3. Both systems can be recommended
   4. I do not know
6. Frequency of change in suction systems
   1. Daily changes are recommended (or when clinically indicated)
   2. Weekly changes are recommended (or when clinically indicated)
   3. It is recommended to change systems for every new patient (or when clinically indicated)
   4. I do not know
7. Endotracheal tubes with extra lumen for drainage of subglottic secretions
   1. These endotracheal tubes reduce the risk of VAP
   2. These endotracheal tubes increase the risk of VAP
   3. These endotracheal tubes do not influence the risk of VAP
   4. I do not know
8. Kinetic vs. standard beds
   1. Kinetic beds increase the risk of VAP
   2. Kinetic beds reduce the risk of VAP
   3. The use of kinetic beds does not influence the risk of VAP
   4. I do not know
9. Patient positioning
   1. Supine positioning is recommended
   2. Semi-recumbent positioning is recommended
   3. The position of the patient does not influence the risk of VAP
   4. I do not know
10. Use of 0.12% chlorhexidine gluconate antiseptic oral rinse
    1. 0.12% chlorhexidine gluconate antiseptic oral rinse reduce the risk of VAP
    2. 0.12% chlorhexidine gluconate antiseptic oral rinse increase the risk of VAP
    3. 0.12% chlorhexidine gluconate antiseptic oral rinse does not influence the risk of VAP
    4. I do not know

**PART II: Self-Reported Adherence and Barriers to the TMC VAP Bundle**

Number:________

*Instructions: Place a check on the corresponding answer*

1. I always comply with the TMCVAP bundle. Yes____ No____

*If no, why not? (encircle all that applies)*

1. Disagreement with reported trial results
2. Inadequate resources
3. Fear of potential adverse effects
4. Costs
5. Patient discomfort
6. Lack of education
7. Lack of guidelines
8. Other reason, what?__________________________________________
9. I interrupt continuous sedative infusions as recommended. Yes____ No____

*If no, why not? (encircle all that applies)*

1. Disagreement with reported trial results
2. Inadequate resources
3. Fear of potential adverse effects
4. Costs
5. Patient discomfort
6. Lack of education
7. Lack of guidelines
8. Other reason, what?__________________________________________
9. I adhere to existing oral care protocol. Yes____ No____

*If no, why not? (encircle all that applies)*

1. Disagreement with reported trial results
2. Inadequate resources
3. Fear of potential adverse effects
4. Costs
5. Patient discomfort
6. Lack of education
7. Lack of guidelines
8. Other reason, what?__________________________________________
9. I always use chlorhexidine oral rinse as recommended. Yes____ No____

*If no, why not? (encircle all that applies)*

1. Disagreement with reported trial results
2. Inadequate resources
3. Fear of potential adverse effects
4. Costs
5. Patient discomfort
6. Lack of education
7. Lack of guidelines
8. Other reason, what?__________________________________________
9. I always perform subglottic suctioning as recommended. Yes____ No____

*If no, why not? (encircle all that applies)*

1. Disagreement with reported trial results
2. Inadequate resources
3. Fear of potential adverse effects
4. Costs
5. Patient discomfort
6. Lack of education
7. Lack of guidelines
8. Other reason, what?__________________________________________
9. I always use closed suction system for all my patients. Yes____ No____

*If no, why not? (encircle all that applies)*

1. Disagreement with reported trial results
2. Inadequate resources
3. Fear of potential adverse effects
4. Costs
5. Patient discomfort
6. Lack of education
7. Lack of guidelines
8. Other reason, what?__________________________________________
9. I assess the depth of sedation as often as recommended. Yes____ No____

*If no, why not? (encircle all that applies)*

1. Disagreement with reported trial results
2. Inadequate resources
3. Fear of potential adverse effects
4. Costs
5. Patient discomfort
6. Lack of education
7. Lack of guidelines
8. Other reason, what?__________________________________________
9. I interrupt continuous sedative infusions as recommended. Yes____ No____

*If no, why not? (encircle all that applies)*

1. Disagreement with reported trial results
2. Inadequate resources
3. Fear of potential adverse effects
4. Costs
5. Patient discomfort
6. Lack of education
7. Lack of guidelines
8. Other reason, what?__________________________________________
9. I asses the depth of sedation using validated tool. Yes____ No____

*If no, why not? (encircle all that applies)*

1. Disagreement with reported trial results
2. Inadequate resources
3. Fear of potential adverse effects
4. Costs
5. Patient discomfort
6. Lack of education
7. Lack of guidelines
8. Other reason, what?__________________________________________
9. I perform spontaneous breathing test as recommended. Yes____ No____

*If no, why not? (encircle all that applies)*

1. Disagreement with reported trial results
2. Inadequate resources
3. Fear of potential adverse effects
4. Costs
5. Patient discomfort
6. Lack of education
7. Lack of guidelines
8. Other reason, what?__________________________________________
9. I always keep head of bed elevated to 30-45 degrees. Yes____ No____

*If no, why not? (encircle all that applies)*

1. Disagreement with reported trial results
2. Inadequate resources
3. Fear of potential adverse effects
4. Costs
5. Patient discomfort
6. Lack of education
7. Lack of guidelines
8. Other reason, what?__________________________________________
9. I always make sure that mechanical DVT prophylaxis are inserted as recommended.

Yes____ No____

*If no, why not? (encircle all that applies)*

1. Disagreement with reported trial results
2. Inadequate resources
3. Fear of potential adverse effects
4. Costs
5. Patient discomfort
6. Lack of education
7. Lack of guidelines
8. Other reason, what?__________________________________________
9. I always give pharmacological DVT prophylaxis, as recommended. Yes____ No____

*If no, why not? (encircle all that applies)*

1. Disagreement with reported trial results
2. Inadequate resources
3. Fear of potential adverse effects
4. Costs
5. Patient discomfort
6. Lack of education
7. Lack of guidelines
8. Other reason, what?__________________________________________

**Supplemental Appendix 2:** **VAP Bundle Compliance Checklist**

| **VAP BUNDLE COMPLIANCE CHECK LIST** | | |
| --- | --- | --- |
| **Bed No___________ Shift: AM_______ PM______** | | |
| ***HEAD OF BED ELEVATION***  *Position a protractor with the x-axis situated at the base of the bed, then measure the angle of the head of the bed.*  *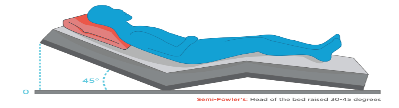* | - *YES (within 30-45 degrees)* | - *NO (not within 30-45 degrees)* |
| ***ASPIRATION OF SUBGLOTTIC SECRETIONS***  *Check the nurses’ monitoring sheet if subglottic secretions were suctioned.* | - *YES, subglottic secretions were suctioned* | - *NO, subglottic secretions were not suctioned* |
| ***CLOSED SUCTIONING SYSTEM***  *Check if a closed suction system is attached to the endotracheal tube, and inquire with patient’s NIC if there was any instance during his/her shift if an open system was used.* | - *YES, closed suction system is attached and was used exclusively during entire shift* | - *NO, closed suction system is not available* - *NO, open suction was used at any time during the shift*   *(Check all that applies)* |
| ***DAILY ASSESSMENT OF READINESS TO EXTUBATE***  *Check the chart if there were any attempts that day to wean patient from the mechanical ventilator (ie, shift to SIMV or CPAP mode), whether or not it was successful.* | - *YES, patient was assessed for readiness to extubate this day.* | - *NO, patient was not assessed for readiness to extubate this day.* |
| ***DVT Prophylaxis***  *If using pharmaceutical prophylaxis, check if the due dose was given during that shift or has been completed by the other shift already for that day.*  *If using continuous infusion, check if the drip is running.*  *If using mechanical prophylaxis, check if the device is in place or was used earlier.* | - *YES, DVT prophylaxis was given.* | - *NO, DVT prophylaxis was not given.* |
| ***ORAL CARE***  *Check medication record if oral antiseptic (ie0.12% Chlorhexidine antiseptic) was used during the shift.* | - *YES, oral care was done.* | - *NO, oral care was not done.* |
| ***COLOR CODING***  *Check the tubings of the mechanical ventilator if the color code is attached and if it is still within its acceptable use date.* | - *YES, color coding is being used and properly implemented.* | - *NO, color coding is NOT being used and/or NOT being properly implemented.* |
